# Trends in clinical workload in UK primary care 2005–2019: a retrospective cohort study

**DOI:** 10.3399/BJGP.2023.0527

**Published:** 2024-09-03

**Authors:** Lyvia de Dumast, Patrick Moore, Kym IE Snell, Tom Marshall

**Affiliations:** Institute of Applied Health Research, University of Birmingham, Birmingham.; Population Health Sciences, University of Bristol, Bristol Medical School, Bristol.; Institute of Applied Health Research, University of Birmingham; National Institute for Health and Care Research, Birmingham Biomedical Research Centre, Birmingham.; Institute of Applied Health Research, University of Birmingham, Birmingham.

**Keywords:** consultation, morbidity, primary care, staff workload, electronic health records, retrospective study

## Abstract

**Background:**

Substantial increases in UK consulting rates, mean consultation duration, and clinical workload were observed between 2007 and 2014. To the authors’ knowledge, no analysis of more recent trends in clinical workload has been published to date. This study updates and builds on previous research, identifying underlying changes in population morbidity levels affecting demand for primary health care.

**Aim:**

To describe the changes in clinical workload in UK primary care since 2005.

**Design and setting:**

Retrospective cohort study using GP primary care electronic health records data from 824 UK general practices.

**Method:**

Over 500 million anonymised electronic health records were obtained from IQVIA Medical Research Data to examine consulting rates with GPs and practice nurses together with the duration of these consultations to determine total patient-level workload per person–year.

**Results:**

Age-standardised mean GP direct (face-to-face and telephone) consulting rates fell steadily by 2.0% a year from 2014 to 2019. Between 2005 and 2019 mean GP direct consulting rates fell by 5.8% overall whereas mean workload per person–year increased by 25.8%, owing in part to a 36.9% increase in mean consultation duration. Indirect GP workload almost tripled over the 15 years, contributing to a 48.3% increase in overall clinical workload per person–year. The proportion of the study population with ≥3 serious chronic conditions increased from 9.7% to 16.1%, accounting for over a third of total clinical workload in 2019.

**Conclusion:**

Findings show sustained increases in consulting rates, consultation duration, and clinical workload until 2014. From 2015, however, rising demand for health care and a larger administrative workload have led to capacity constraints as the system nears saturation.

## Introduction

Strong primary care is associated with better population health, lower healthcare expenditure, and a more equitable distribution of health resources.^1^ In the UK, primary care plays an essential role in the provision of health care, accounting for approximately 90% of all NHS contacts.^2^ Although NHS activity data indicate that general practices delivered a record 356 million appointments in 2023, demand continues to outstrip capacity.^3^ A recent survey reported that 71% of GPs in the UK found their job to be very or extremely stressful, with the highest proportion among the 10 high-income countries surveyed.^4^

Fears that primary care in the UK is in crisis or nearing breaking point are nothing new.^5^ Although pressures on general practice were undeniably exacerbated by the COVID-19 pandemic, the current situation is the outcome of many years of underinvestment, a shrinking of the GP workforce, an ageing and growing population, and national strategic objectives that sought to shift care out of hospitals and into the community. Analysis of 2023 workforce data showed an 11.8% fall in the number of full-time equivalent (FTE) GPs (excluding locums, trainees, and retainers) and a 41% increase in the number of patients per FTE GP since 2014.^1^^,^^6^ The proportion of NHS funding directed to general practices declined from 10.6% in 2005/2006 to 6.8% in 2020/2021 as secondary care services secured a greater share of increases in healthcare spending.^7^ The UK population increased by 11.4% over the same period and its median age rose from 38.7 to 40.7 years.^8^

Analysis of a large database of electronic health records described a 10.5% increase in annual consultation rates per person between 2007 and 2014, mainly accounted for by an increase in GP consultations.^9^ The same period also saw an increase in consultation duration. In cross-sectional analysis, consultation rates were higher in older patients, females, and those living in more deprived regions.^10^ A similar analysis of duration found GP consultations were longer in older patients and females, although the differences were small.^11^ The focus of much of the literature on GP workload is on direct patient care, an activity that typically accounts for 75% of patient-related clinical workload.^12^ Time spent on indirect patient care (for example, referral letters or repeat prescriptions) was not included, implying that primary care workload data may under-represent total patient-related clinical activity by a third.

**Table:** How this fits in

Previous literature on GP and practice nurse face-to-face or telephone consultations showed an increase in direct patient workload between 2007 and 2014. This study examines all aspects of patient workload, both direct contacts and patient-related administrative work, in terms of consulting rates per person–year and the duration of these consultations from 2005 to 2019. Health and social care system changes, rising levels of morbidity, and increased demand from patients have all combined to place additional pressures on UK general practice.

Many questions remain unanswered. There is limited understanding of the factors driving long-term trends in consultation rates. The aim of this analysis of the volume and nature of GP and practice nurse consultations was to obtain objective data on changes in clinical workload between 2005 and 2019. Overall clinical workload over time, workload by clinical role, and by multimorbidity level are examined.

## Method

### Study design

A retrospective cohort study was carried out using data obtained from IQVIA Medical Research Data UK (IMRD) incorporating data from The Health Improvement Network, a Cegedim database. IMRD includes anonymised electronic primary health care records from approximately 6% of the UK population in over 800 UK general practices. General practices are largely representative of UK primary care practices in size, age, and the sex of patients, and prevalence of chronic conditions.^13^

Data were extracted for all patients registered with practices contributing to IMRD, covering the period 1 January 2005 to 31 December 2019. Data extraction was facilitated using the Data Extraction for Epidemiological Research (DExtER) tool.^14^

### Outcomes

The primary outcome is individual patient clinical workload, defined as the total number of contact minutes per year that the patient has with their general practice, coded by staff role and by type of contact. A GP contact is defined as any file opening by a GP and includes face-to-face consultations, telephone calls to or from a patient, results recording, or issuance of a repeat prescription. Similarly, a nurse contact is defined as any file opening recorded as being made by a practice nurse. Patient consultations with nurses are mainly separate from those with doctors. In the UK, primary care nurses’ responsibilities include immunisation, cervical screening, health promotion, and chronic disease management.^15^ All non-clinical work by a GP or practice nurse was excluded from the workload calculations, as was any work done by other clerical or administrative staff or other providers of direct care such as physiotherapists or dieticians.

File openings of 0 min have been rounded up to 30 s. File openings of ≥30 min were truncated at 30 min as long openings were considered unlikely to reflect patient work. Consultation rates are defined as the number of times a patient’s file is opened per person–year, by a nurse or a GP. Consultation rates for direct patient contacts (face-to-face surgery consultations and telephone consultations) are also reported. Clinical workload per person–year is defined as the sum of all GP and nurse contact minutes for a given patient in a given year.

### Multimorbidity status

Information about patients’ long-term conditions was obtained from IMRD with medical diagnoses of these conditions recorded using the Read code clinical classification system. Read codes are a hierarchical clinical terminology system used within both primary and secondary care to record a wide range of information relating to a patient’s demography, symptoms, tests, results, and diagnoses.

Previous work by Barnett *et al* identified 40 long-term conditions that had a significant impact on a patient’s quality of life, risk of mortality, and need for health care.^16^ In the current study the code lists associated with each of these conditions as determined by a multimorbidity research joint project between the Universities of Cambridge and Birmingham was used.^17^

Consulting patterns from 2015 until 2019 were examined, comparing individual workload at 1-year pre-diagnosis to workload 1 year, 3 years, and 5 years post-diagnosis for each condition to determine the length of time that conditions should be shown as present following diagnosis.

### Analysis

Person–years for each age group were calculated for each year. Workload per person–year and consultation rates were age standardised to the population of the 2005 IMRD dataset to allow comparison over time. Mean annual clinical consulting rates and mean duration of file openings were calculated for all types of consultations with a GP, face-to-face and telephone consultations with a GP, and consultations with a practice nurse. Patients were grouped according to how many chronic conditions they had (0, 1, 2, and ≥3 conditions) and average workload per person–year calculated for each group over the period. Summary statistics are presented in the following section, either graphically or in tables.

## Results

Overall, data for over 550 million file openings for 10 098 454 patients from 824 practices were examined in this study, representing over 69 million person–years of observation. Descriptive statistics are given for 2005 and 2019 (Table 1).

**Table 1.** Descriptive statistics of dataset

**Characteristic**	**2005, *n* (%)**	**2019, *n* (%)**
**Patients, *n***	5 159 933	2 785 796

**Number of person–years**	4 885 863	2 604 468

**Sex, %**		
Male	2 416 088 (46.8)	1 290 586 (46.3)
Female	2 743 845 (53.2)	1 495 210 (53.7)

**Age group, years, %**		
0–4	297 698 (5.8)	128 739 (4.6)
5–14	524 473 (10.2)	306 250 (11.0)
15–44	2 039 569 (39.5)	964 178 (34.6)
45–64	1 368 371 (26.5)	780 224 (28.0)
65–74	486 507 (9.4)	323 769 (11.6)
75–84	324 643 (6.3)	199 228 (7.2)
≥85	118 672 (2.3)	83 408 (3.0)

**Age, years, mean (SD)**	41.7 (23.1)	43.9 (23.8)

**Multimorbidity**		
Number of conditions, mean (SD)	0.9 (1.2)	1.2 (1.4)
With 0 serious conditions	2 659 429 (51.5)	1 215 068 (43.6)
With 1 serious condition	1 339 519 (26.0)	691 589 (24.8)
With 2 serious conditions	660 987 (12.8)	432 079 (15.5)
With ≥3 serious conditions	499 998 (9.7)	447 060 (16.1)

**Practices, *n***	824	403

**Size of practice, mean (SD)**	6262 (3453)	6836 (3613)

*SD = standard deviation.*

A comparison of the population by age group for the dataset and for the UK population as a whole in 2005 and 2019 shows that the sample is broadly similar to UK national data obtained from the World Bank databank.^18^ For example, in 2005, 59.7% of the UK population was aged <45 years compared with 55.5% for the sample (*n* = 2 861 740/5 159 933). In 2019, 55.7% of the UK population was aged <45 years compared with 50.2% for the sample (*n* = 1 399 167/2 785 796) (see Supplementary Table S1).

### GP face-to-face/telephone consulting rates

After an initial drop in the age-standardised mean consulting rate, rates climbed to a high of 3.84 (95% confidence interval [CI] = 3.84 to 3.85) direct consultations per year in 2014. From 2014 mean consulting rates fell steadily by 2.0% a year to 3.47 (95% CI = 3.46 to 3.47) consultations per year by 2019. Between 2005 and 2019 mean consulting rates fell by 5.8% overall (see Supplementary Figure S1).

### Duration of file openings

Duration of file openings by practice nurses increased at a relatively constant rate over the period from a mean of 6.83 (95% CI = 6.82 to 6.83) min in 2005 to 8.99 (95% CI = 8.98 to 9.00) min in 2019, a rise of 31.7% overall (see Supplementary Figure S2).

For GP face-to-face or telephone consultations, mean duration increased by 36.0% between 2005 and 2011. From 2011 onwards, the rate of increase in mean duration of GP face-to-face consultations plateaued, remaining between 8.21 (95% CI = 8.21 to 8.21) min and 8.46 (95% CI = 8.45 to 8.46) min until 2019. The biggest change was in all GP file openings where mean duration increased by 68.4% from 4.57 (95% CI = 4.57 to 4.57) min in 2005 to 7.69 (95% CI = 7.69 to 7.70) min by 2019 (see Supplementary Figure S2). From 2005 to 2019, mean duration of GP direct consultations increased by 36.9% overall.

### Clinical workload

**Figure 1.**
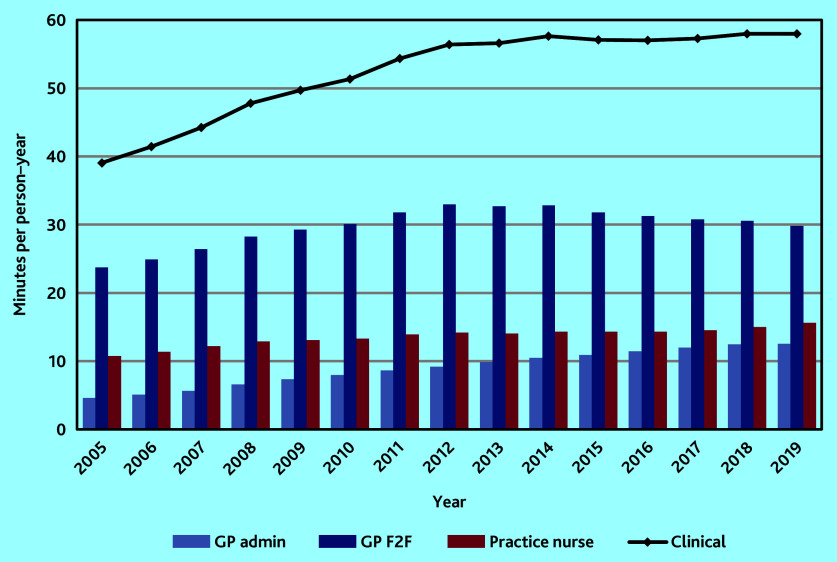
Mean age-standardised workload per person–year by staff role. F2F = face to face.

Age-standardised mean clinical workload per person–year increased by over 48% from 39.06 (95% CI = 39.03 to 39.10) min in 2005 to 57.61 (95% CI = 57.55 to 57.66) min in 2014. From 2014 to 2019 it remained relatively stable, fluctuating between 56.98 (95% CI = 56.93 to 57.03) and 57.98 (95% CI = 57.93 to 58.03) min (Figure 1).

### GP workload

In the study, GP workload was separated into two parts: patient-facing workload (GP F2F: all face-to-face consultations and telephone consultations) and patient-related administrative work (GP admin). Mean GP F2F workload per person–year increased every year between 2005 and 2012 to a maximum of just under 33 min. From 2012 to 2019 it fell by 10.6% to just below 30 min. Mean GP admin workload stood at 4.60 (95% CI = 4.60 to 4.60) min per person–year in 2005 rising to 12.53 (95% CI = 12.52 to 12.55) min by 2019, an increase of 172.4%. Administrative workload as a proportion of total GP workload nearly doubled from 16.3% in 2005 to 29.6% in 2019 (Figure 1).

### Practice nurse workload

Age-standardised mean practice nurse workload per person–year rose consistently over the period from 10.75 (95% CI = 10.74 to 10.76) min in 2005 to 15.58 (95% CI = 15.56 to 15.59) min in 2019, an increase of 44.9% overall (Figure 1).

Changes over the period in age-standardised mean workload by staff role and type of consultation are shown in Table 2.

**Table 2.** Age-standardised mean workload (in minutes per person–year) by staff role

**Staff role**	**2005**	**2019**	**% change (95% CI)^a^**	**% change/year^a^**
**Practice nurse**	10.75	15.58	44.9 (44.6 to 45.1)	2.7
**GP F2F**	23.71	29.83	25.8 (25.6 to 26.0)	1.7
**GP admin**	4.60	12.53	172.4 (171.9 to 172.9)	7.4
**GP all**	28.31	42.36	49.6 (49.3 to 49.9)	2.9
**Clinical**	39.06	57.94	48.3 (48.0 to 48.6)	2.9

a *Percentage change calculated as: ((workload at end of period – workload at start of period)/workload at start of period) × 100. Percentage change per year calculated as: ((workload at end of period/workload at start of period)^1/n^ – 1) × 100, where* n *= 14 (number of years from 2005 to 2019). Clinical = sum of nurse and GP workload. GP admin = indirect patient-related workload. GP all = indirect + direct patient workload. GP F2F = face-to-face/telephone consultations with a GP.*

### Multimorbidity levels

Analysis of the impact of a diagnosis on workload found that for most conditions clinical consultation time increased considerably in the year of diagnosis compared with the year before diagnosis, however, consultation time returned to below pre-diagnosis levels within 5 years. For 11 conditions, consultation time increased considerably in the year of diagnosis and remained at a higher level even after 5 years. These conditions were coded to show as present indefinitely, whereas all the other conditions were coded to show as present for 5 years only following diagnosis (see Supplementary Information S1 for details).

**Figure 2.**
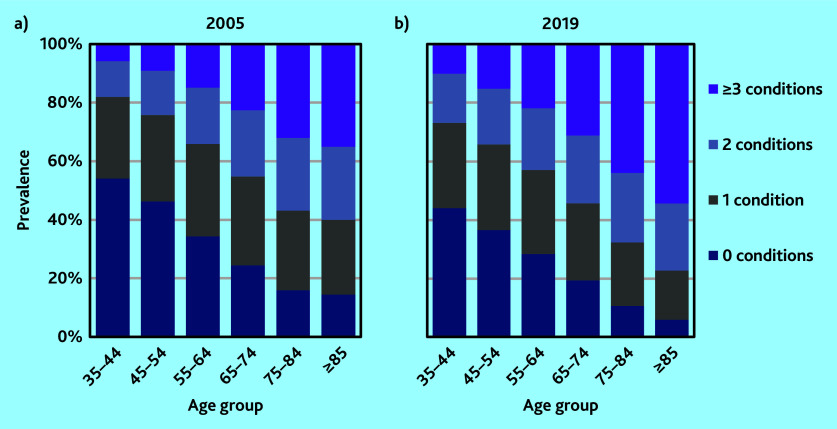
Prevalence of chronic conditions by age group. a) 2005; and b) 2019.

Multimorbidity increased across all older age groups between 2005 and 2019 (Figure 2). Overall, 51.5% of the study population had no serious chronic conditions recorded in 2005 and accounted for 27.9% of total clinical workload. Patients with multimorbidity with ≥3 serious chronic conditions represented just 9.7% of the study population but 24.2% of the workload (*n* = 499 998/5 159 933). By 2019 the share of the population without any serious chronic conditions had fallen to 43.6% whereas that for patients with multimorbidity with ≥3 conditions had increased to 16.0% (*n* = 447 060/2 785 796). The share of total clinical workload accounted for by these patients was 34.5%.

**Figure 3.**
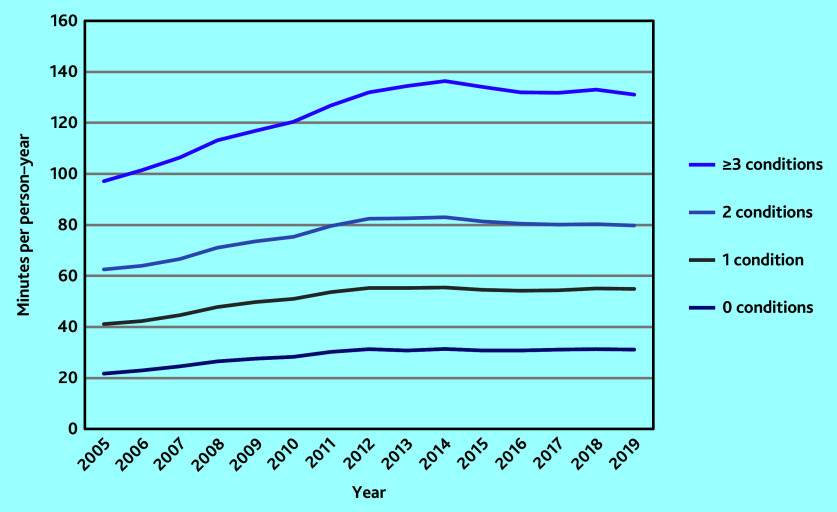
Clinical workload by number of chronic conditions.

The mean clinical workload associated with patients with no chronic conditions was 21.71 (95% CI = 21.67 to 21.75) min in 2005. Clinical workload increased linearly with the number of chronic diseases: mean workload was 41.13 (95% CI = 41.04 to 41.22) min for patients with one condition, 62.54 (95% CI = 62.37 to 62.71) min for two conditions, and 97.14 (95% CI = 96.86 to 97.42) min for ≥3 conditions. In 2019, mean workload was 31.08 (95% CI = 30.99 to 31.16) min, 54.83 (95% CI = 54.67 to 54.99) min, 79.73 (95% CI = 79.45 to 80.00) min, and 131.03 (95% CI = 130.63 to 131.42) min for 0, 1, 2, and ≥3 conditions, respectively (Figure 3).

The rate of increase in clinical workload per person–year over the study period was highest for patients with no chronic conditions at +43.2%, compared with a +33.3% increase in workload for those with one condition, +27.5% for two conditions, and +34.9% for ≥3 conditions (Figure 3).

## Discussion

### Summary

This study examined trends in consulting rates and duration of consultations for GPs and practice nurses from 2005 to 2019. To capture the full scope of patient-level activity, all aspects of GP workload were studied: both time spent in face-to-face and telephone consultations as well as patient-related administrative work, such as results recording or third-party consultations. Direct patient workload has considerably increased over the period for both GPs and practice nurses by roughly the same amount. However, the amount of time spent by GPs doing patient-related administrative work has increased enormously.

Many factors are likely to have contributed to the increased admin workload of GPs observed over the study period, including the increased ability of GPs to access diagnostic services directly, the transfer of work from secondary to primary care, as well as the introduction of the Quality and Outcomes Framework (QOF) in 2004.^19^^,^^20^ The QOF is a pay- for-performance scheme intended to reward primary care providers for improvements in the management of long-term conditions, representing over 8% of total practice income on average in 2019/2020.^21^ Little existing literature on the QOF examines its impact on administrative work undertaken by GPs. There is evidence to suggest, however, that its introduction led to a substantial increase in non-consultation GP workload, in particular that associated with tests. A study of changes in diagnostic testing in UK primary care reported a 3.3-fold increase in test use between 2000/2001 and 2015/2016, and estimated that the average GP spent 1.5 to 2 h each day reviewing test results.^22^

The current study recorded a plateauing of the rate of increase in clinical workload from 2014 onwards, with the higher levels of GP admin workload making up for the decline in the GP face-to-face or telephone consulting rate.

### Strengths and limitations

The main strength of this study is that it is the first, to the authors’ knowledge, to report on trends in overall clinical workload, examining duration and frequency of clinical consultations, for both patient-facing and administrative activity related to a direct patient contact, such as a repeat prescription or recording of test results. Its findings are based on nearly 70 million person–years of observation covering a 15-year period for practices throughout the UK, making it, to the authors’ knowledge, the largest analysis of clinical workload to date.

This study has several limitations. The most important limitation is that it was not possible to include data from 2020 onwards in the analysis. However, it was felt that the considerable disruption in primary care use during the COVID-19 pandemic was unlikely to be permanent and consequently that the use of data from that period and shortly after would not be representative of any underlying trend. Although the IMRD database is one of the most comprehensive data sources worldwide, as is the case for many observational studies using electronic health records, the accuracy of the recording of consultation durations and types is variable. Short file openings for face-to-face consultations may not accurately reflect the actual work associated with a particular patient if the practitioner does not open the file at the beginning of a consultation, underestimating workload. Similarly, workload will be overestimated if a practitioner forgets to close a patient’s file at the end of the session (all consultations were truncated at 30 min to mitigate this problem).

The list of chronic conditions selected to determine morbidity levels is based on highly regarded previous work: the Read codes used to define these conditions for the present study closely mirror those used by Barnett *et al* and Cassell *et al* but may differ slightly.^16^^,^^23^ Using a different set of conditions may have given different results in terms of prevalence and workload associated with the different levels of multimorbidity.

### Comparison with existing literature

This study supports previous literature that showed an increase in face-to-face and telephone GP and practice nurse workload between 2007 and 2014 in English general practices, observing both a rise in the mean number of consultations per year and a 4.9% increase in consultation duration.^9^ Research by Kontopantelis *et al* described an increase in the number of GP consultations per year from a median of 5.3 to 8.3 between 2000 and 2019, whereas the number of face-to-face GP consultations per year per patient fell from 3.7 to 3.1.^24^ Analysis of the use of primary care by children in England reported a fall in general practice consulting rates of 1% per year in all age bands (except for infants) between 2007 and 2017 while observing a corresponding rise in urgent care use.^25^

Whereas literature examining overall trends in clinical workload is scarce, considerable research has examined the association between primary care use and multimorbidity. The crude prevalence rate of multimorbidity (defined as the presence of ≥2 long-term conditions) was 31.6% in the present study in 2019 compared with 22.5% in 2005, rates that are broadly consistent with previous studies of similar populations in the UK.^16^^,^^23^ Multimorbid patients consulted a GP 2.6 times more frequently^23^ and each consultation lasted 0.2 min longer on average than for patients without multimorbidity.^26^ Using a different definition of multimorbidity (≥2 chronic conditions of the 17 conditions included in the QOF), the first comprehensive study published on the prevalence of morbidity in England identified 16% of patients as being multimorbid in 2008 and these patients accounted for almost a third of all primary care consultations. Patients with multimorbidity had on average 9.4 consultations per annum compared with 3.8 for those without multimorbidity.^27^

### Implications for research and practice

Primary care practices have had to adjust to consistent increases in the duration of nurse and GP contacts since 2005 in the face of higher numbers of patients with multimorbidity with complex care needs and a greater administrative load per patient. With fewer FTE GPs per head of population, many practices have been unable to keep pace with these changes, leading to a drop in consulting rates since 2015.

The implications of this for practice funding and access to care are important. Approximately half of practice revenue is from the global sum payment, with the amount allocated based on an estimate of a practice’s patient-level workload using demographic data that is over 20 years old. The statistical model used is commonly known as the Carr-Hill formula and it includes factors relating to patient age and gender, morbidity and mortality measures, the number of newly registered patients, staff expenses, practice rurality, and the number of patients living in nursing and residential homes. It is widely recognised that the Carr-Hill formula does not adequately reflect population healthcare needs, particularly need associated with socioeconomic deprivation.^28^^,^^29^ Previous research reported that practices in areas of greater deprivation received 7% less funding per need-adjusted patient than those in more affluent areas.^30^ An analysis of primary care funding in England for 2015–2016 found only a modest association between practice funding and morbidity burden at the regional level, with the North East and North West regions appearing to be particularly under-resourced.^28^

Repeated calls on the government to replace the Carr-Hill formula with a more equitable formula that better reflects the greater workload associated with deprivation and morbidity have resulted in little progress. Acknowledging in 2015 that the current formula is ‘out of date and needs to be revised’, NHS England and the British Medical Association committed to review the Carr-Hill formula, anticipating that the work would be completed by the summer of 2016.^31^ The timeline for reporting findings has since been extended several times but no details of any proposed changes to the formula have been reported to date.

## Data

The IMRD-UK dataset cannot be shared under the data-sharing agreement with the University of Birmingham on behalf of IQVIA.
